# Global neurosurgery needs reciprocity: bidirectional exchange as a workforce strategy

**DOI:** 10.1016/j.bas.2026.106122

**Published:** 2026-06-08

**Authors:** KCH-UDMNINAS Neurosurgical Collaborative, Prajwal Ghimire, Difei Wang, Jose Lavrador, Francesco Vergani, Bassel Zebian, Richard Gullan, Pedro Coelho, James Heritage, Dipendra Yadav, Ram Subedi, Sagar Koirala, Suresh Bishwokarma, Suraj Thulung, Pratyush Shrestha, Nikunj Yogi, Milan Gurung, Madhu Devkota

**Affiliations:** a King's College Hospital NHS Foundation Trust, London, UK; b Neurophys. Ltd, UK; c NICO Corp, United States; d Aberdeen Royal Infirmary, Scotland, UK; e Upendra Devkota Memorial National Institute of Neurological and Allied Sciences (UDM-NINAS) and Upendra Devkota Foundation (UD Foundation), Kathmandu, Nepal

**Keywords:** Global neurosurgery, Sustainable workforce, Bidirectional learning, Sustainable model

Exchange of neurosurgical skills has a potential in addressing the unmet need of tackling operable neurosurgical pathologies between High-income countries (HICs) and Low-middle-income countries (LMICs) which form a major part of the brain-health-related conditions in both settings despite differences in epidemiology (World Health Organization, 2022; Ostrom et al., 2019; Tamimi et al., 2017; RCSEd Strategy).

We are excited to share our initiative of bilateral neurosurgical skills transfer between Nepal and United Kingdom identifying the unrealized potential of bidirectional knowledge exchange.

In partnership with the Upendra Devkota Foundation, a multidisciplinary UK faculty delivered a minimally invasive neurosurgical cadaveric simulation course and a national neuro-oncology workshop alongside subspecialty conference teachings in Nepal. Over 60 neurosurgeons and trainees participated across programmes. A SBNS-funded (SBNS Global Observership) six-week UK clinical observership facilitated exposure to advanced operative workflows, perioperative safety systems, and structured multidisciplinary care.

Following these bidirectional programmes, a mixed-method structured feedback was collected from ten faculty across both institutions. All ten respondents agreed that the collaboration promoted mutual rather than unidirectional learning (mean score 4.6/5.0) and was appropriate to local clinical context (4.7/5.0). Nine of ten agreed the programme would improve patient care pathways and workforce development. Eight of ten reported plans to change practice, with key areas including minimally-invasive operative approaches, intraoperative neurophysiology, multidisciplinary decision-making, and perioperative safety. All respondents endorsed international expansion of this model (4.7/5.0).

We, thus, propose a reciprocal global neurosurgical education model emphasizing a bidirectional learning encompassing knowledge sharing and skills exchange (Fig. 1). Global neurosurgery partnerships should be structured around reciprocal institutional frameworks rather than episodic outreach. A practical model would include joint needs-and-strengths mapping, co-designed educational exchange, context-sensitive implementation of transferable practices, and prospective evaluation of workforce and service impact. Such partnerships should then progress towards recurring simulation courses, observerships, fellowship pathways, and joint academic programmes, providing a scalable route to sustainable workforce development in both low-resource and high-resource settings.

**Fig. 1:**
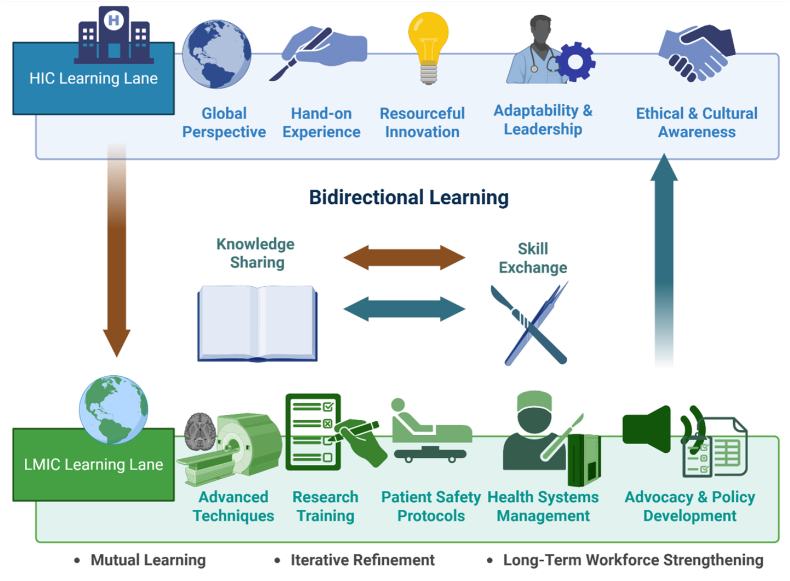
Reciprocal neurosurgical education model for bidirectional learning. The HIC learning lane includes understanding the global perspective, hands-on experience on regional neurosurgical conditions and techniques, resourceful innovation techniques, adaptability & leadership skills and ethical & cultural awareness. On the other hand, the LMIC learning lane includes advanced techniques, clinical and academic research training, advancement of patient safety protocols, non-technical skills related to health systems management and policy development. This model reinforces mutual learning, iterative refinement of skills and long-term workforce strengthening with foundations of knowledge sharing and skills exchange. (Created in Biorender.com).

## Authors’ contribution

KCH-UDMNINAS Neurosurgical Collaborative∗: Conceptualization, Methodology, Project administration, Supervision, Writing – original draft, Writing – review & editing.

## Ethics approval and consent to participate

No patient data was utilized in the study. Consent to participate is not applicable.

## Consent for publication

Not applicable.

## Availability of data and material

Not applicable.

## Funding

Not applicable.

## Competing interest

Not applicable.
